# A Curvature Sensor Utilizing the Matteucci Effect in Amorphous Wire

**DOI:** 10.3390/s23031243

**Published:** 2023-01-21

**Authors:** Sahar Alimohammadi, Paul Ieuan Williams, Turgut Meydan

**Affiliations:** Cardiff School of Engineering, Cardiff University, Queen’s Buildings, Cardiff CF24 3AA, UK

**Keywords:** Matteucci effect, flexible bending sensor, amorphous wire

## Abstract

The study of wearable sensors for human disease monitoring has developed into an important research area due to its potential for personalized health care. Various sensor types have been proposed for assessing the range of joint movement in patients with progressive diseases or following post-surgical treatments. Many of these methods suffer from poor accuracy, sensitivity, and linearity or are very expensive and complex to implement. To overcome some of these limitations, this paper reports on the development of a novel flexible sensor for the measurement of bending by utilizing the Matteucci effect in the amorphous wire. This paper describes a bend sensor that utilizes positive magnetostrictive amorphous wire to achieve a measurement sensitivity equal to 5.68 ± 0.02 mV/cm with a resolution of ±0.2° over a measuring range of 64 to 143°.

## 1. Introduction

In recent years demand for wearable sensors has increased significantly in many application areas such as medical, entertainment, security, and military. Flex sensors are the most prevalent type in modern wearable devices, particularly in the area of instrumented gloves used for measuring hand and finger posture. These gloves, however, were primarily developed for use in applications such as virtual reality and digital gaming and do not meet the exacting standards required for clinical use. The potential for wearable devices is significant, diseases such as Dupuytren’s contracture, which limits fine motor control and movement, affect twenty percent of adults over the age of 65 in the UK [1]. A wearable device offers the potential to assess hand motor function or post-operative healing more regularly away from the clinic, which is essential for continuous health monitoring.

Flex sensors, also known as bend sensors or angular displacement sensors, measure the degree of bending as a change in some property such as resistance, voltage, capacitance, or light intensity. Early flex sensors were fabricated by coating or printing carbon/polymer ink-based materials on to flexible plastic substrates such as polyimide or polyester [2]. They were initially adopted in home entertainment devices as part of the “Power Glove” motion control system (Abrams) [3]. A year later its production ceased due to its lack of precision and difficulty of operation [4]. Several manufacturers including Images Scientific Instruments Inc [5], Spectra Symbol Crop [6], and Flexible Point Sensor System Inc, (Flexpoint SS) [7] have produced examples of early commercial bend sensors.

**Figure 1:**
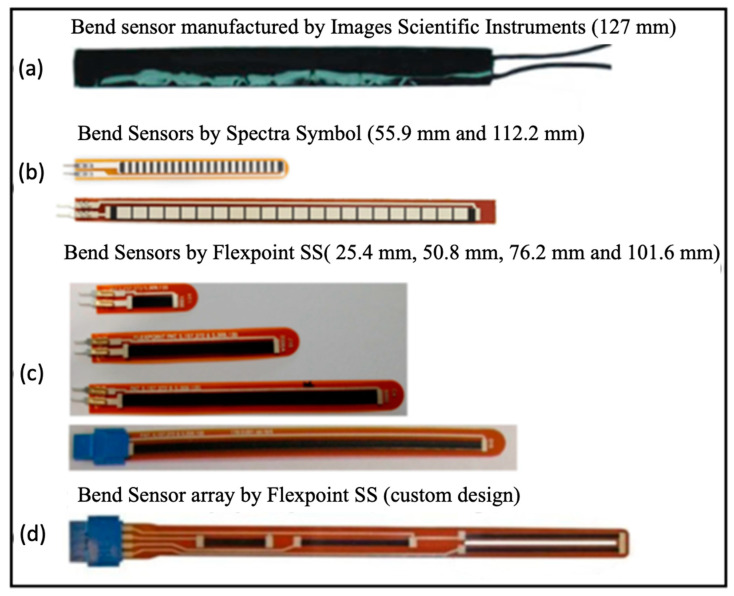
Images of commercial bend sensors (**a**) bi-directional bend sensors manufactured by [5]; (**b**) bend sensors produced by Spectra Symbol [12]; (**c**) bend sensors with different lengths manufactured by Flexpoint SS [12]; (**d**) custom design, bend sensor array produced by Flexpoint SS [14].

Figure 1a shows a bi-directional bend sensor with a length of 127 mm consisting of a strip of resistive material sandwiched between two copper foil laminates. According to the manufacturer’s specification, the sensor has a nominal resistance of 20 kΩ which decreases gradually by bending in either direction [5]. Orengo [8] developed a piezoresistive model and applied it to commercial bend sensors, showing a linear increase in resistance from 4 kΩ to 16 kΩ for bending angles ranging from 30 to 180°. Although these bend sensors are used in glove sensing systems [9,10,11], they have been discontinued since 2010 because of their low accuracy and stability. The off-the-shelf bend sensors manufactured by Spectra Symbol are usually designed with lengths of 55.9 mm or 112.2 mm, as shown in Figure 1b and exhibit a linear dependence with bending angle. However, a hysteresis error of 14% was reported for the 112.2 mm sensor during bending and unbending operations from 0 to 90°, much higher than that of bend sensors from Flexpoint SS (about 3%) [12]. Simone et al. [13] bent 112.2 mm sensors over a 7.62 cm (3 inch) diameter tube and after 30 s on the tube, observed a 31.8% and 8.9% decay of initial resistance for Spectra Symbol and Flexpoint SS sensors, respectively. Comparing the change in sensor resistance, the Spectra Symbol varied from 8.2 kΩ to 13.1 kΩ when the sensor was bent from 0 to 90° compared with Flexpoint SS resistance variation in 112 kΩ for the same bending range [12]. Overall, the Flexpoint SS sensors were found to be more stable and sensitive to mechanical deformation than the Spectra Symbol bend sensors.

A possible means of improving stability is by the use of a polymer overlay. Borghetti et al. [12] reported that the resistance of polyimide, polyester, and bare bend sensors varied by about 2%, 3%, and 30%, respectively, after bending at 90° for 180 s. Therefore, the over-coated sensors with polyester or polyimide are generally more stable than bare bend sensors.

Flexpoint SS sensors have become one of the most popular sensors used in applications for tracking finger motion [2,15,16,17]. Another sensor based on resistance change is Jurgens et al.’s [18] low-cost sensor produced by screen printing a carbon-based electrically conductive ink on a polyester substrate. The sensor repeatability was 6.5% with a 100 Ω variation in resistance when bending from 0 to 90°. The strengths of carbon-based flex sensors include high flexibility, easy to operate, and low cost. Their limitations are a time-consuming calibration process, large hysteresis errors and failure to return to the initial state after loading, slow response, temporal drift, and suffering from significant overshoot in high-speed situations.

A more sophisticated approach to bend measurement utilizing strain gauges has been achieved using nano-based material structures. An example of this is Rogers et al.’s work on arrays using assemblies of single-crystalline silicon nanoribbons [19]. They achieved a gauge factor of 43 with good repeatability, stability, and high sensitivity. It was proposed that this concept can be used with stretchable or curvilinear substrates for strain mapping in medical applications. Alaferdov et al. [20] developed a simple and scalable method for the fabrication of wearable strain and bend sensors based on high aspect ratio graphite nanobelts thin films deposited by a modified Langmuir–Blodgett technique on to flexible polymer substrates. The sensing mechanism is based on the changes in contact resistance between individual nanobelts due to substrate deformation. Very high stability over 5000 strain release cycles was achieved with a low power consumption of 1 nW. The maximum strain that could be applied to the system was 40%. Other examples of bend sensors include a Hydrazine-Reduced Porous Graphene, for Human Motion Monitoring [21], with a sensitivity of 1.092 mV/°, excellent linearity and a resolution of 1° and a wearable strain sensor based on flexible rubrene single crystal [22] with a high sensitivity gauge factor (GF) of 279.

Other sensing methods that have been investigated include capacitive-based strain sensors, for example, Atalay’s stretchable, conductive-knit fabric within a silicone elastomer matrix for recording knee movement [23]. The linearity of this sensor was 0.997 and a resolution of 1.36%.

Systems based on fiber optics, including hetero-core fibers [24], have been reported for bend measurements but these are limited by their temperature dependence, are relatively expensive, bulky, and difficult to implement as a wearable device. A review of fiber optic sensors [25] as curvature sensors also points out that they are prone to instability and low reproducibility.

Another category of sensors used for bend measurements is the inertial-based measuring devices including accelerometers and gyroscopes. Accelerometers can operate as tilt sensors and provide angle measurements down to 0.02° resolution under controlled conditions [26]. Gyroscopes measure angular velocity and are suited to dynamic measurements. They can be used for angle determination but are subject to integration errors limiting their usefulness. A common approach is to use both gyroscopes and accelerometers combined with a magnetometer, i.e., an inertial measurement unit (IMU) with data fusion algorithms. This approach resolved angle measurements down to 0.007° [27]. Several researchers have reported work on inertial-based systems for measuring body movements [28,29]; however, they are relatively expensive and difficult to use especially for multiple points of measurement, for example, the finger joints.

Flexible 3D-printed sensors form another category of bend sensor. Kouchakzadeh et al. presented a flexible 3D-printed conductive sensor with piezoresistive properties [30]. It was printed from the filaments of thermoplastic polyurethane (TPU) and acrylonitrile butadiene styrene (ABS) with carbon black elements. Results show a gauge factor of 2–3 with strain ranging from 0 to 0.2. Nassar et al. presented a strain sensor fabricated using simultaneous printing of functional materials and conventional polymer-based material [31]. This sensor was tested under static and dynamic bending conditions and showed a linear response in resistance change and exhibited a gauge factor of 1. This sensor has the potential to produce complex 3D structures with less processing steps than the current state-of-the-art methods to obtain fully embedded integrated sensors and circuits. Further research is needed for more complex shapes.

To summarize, current commercial technologies available for monitoring the bending of human joints tend to be expensive fiber optic solutions, low sensitivity semiconductor strain sensors, bulky 3D-printed sensors, complex inertial systems, or less reliable resistance or capacitance-based sensors. There is a clear need for a new generation of wearable devices with improved accuracy whilst remaining unobtrusive to the user [32]. This research aimed to investigate the possibility of developing a high-sensitivity flexible bending sensor, utilizing the Matteucci effect, capable of monitoring body posture or bending whilst offering performance advantages over current technologies.

Ferromagnetic amorphous wires have attracted significant interest in sensor applications using Giant Magneto-Impedance (GMI) [33], strain, magnetic field, and current sensing [34] due to their remarkable magnetic properties [35]. The unique characteristics of amorphous wire include good flexibility, high fatigue strength, a large Barkhausen jump, and a significant Matteucci effect [36]. The Matteucci effect is the generation of voltage pulses at the ends of a ferromagnetic wire when magnetized axially with an alternating field in the presence of torsional stress. The Matteucci effect occurs in all ferromagnetic materials but is particularly strong in amorphous wires [37] and is highly sensitive to the magnetic field amplitude and frequency, applied stress and torsion, and the wire’s physical dimensions. Fine-tuning one or more of these parameters opens the possibility to optimize the bending and tensile stress sensitivity for specific bending measurement applications [38]. Previously, we demonstrated the effect of tensile stress on the Matteucci effect [39]. In this work, the effect of changing tensile stress due to bending has been exploited in a flexible bending sensor utilizing positive magnetostrictive amorphous wire. A flexible solenoid for magnetizing the amorphous wire was designed specifically for measuring curvature. The output Matteucci voltage was measured over curvatures ranging from 64 to 143° to assess sensor performance in terms of its sensitivity and linearity.

## 2. Sensor Design

**Figure 2:**
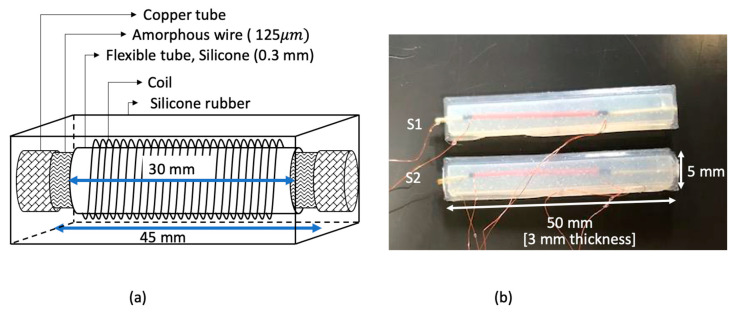
(**a**) Schematic diagram of the bending sensor. (**b**) Flexible bending sensors.

The sensor incorporates Fe_77.5_Si_7.5_B_15_ (AF10) amorphous wire, supplied by Unitika Ltd., with a magnetostriction of $34.5\times 10^{-6}$ [40]. The schematic diagram of the sensor is shown in Figure 2a, the sensing element consists of a 45 mm long, 125 µm diameter amorphous wire placed inside a flexible silicone tube 0.3 mm in diameter and 30 mm long. A solenoid coil with 200 turns of 0.11 mm diameter wire carrying 0.135 A, excites the amorphous wire with a sinusoidal magnetic field of amplitude 0.9 kA/m. The magnetic field was calculated using Equation (1) where *N* is number of turns, I is the current through the coil and l is the wire length.

(1) $$H=\frac{NI}{l}$$

Copper wires were connected across the ends of the amorphous wire to pick up the Matteucci voltage. 

The whole arrangement was embedded inside silicone rubber (Dragon skin 30 from Smooth-on, Inc, Macungie, PA, USA). Silicone rubber was chosen to enable the flexibility of the sensor whilst supporting the coil. The amorphous wire was twisted 40°/cm (271 MPa torsion stress), using a rotation mount, before embedding it in the silicone rubber. The finished dimensions of the sensor were 3 mm thick, 5 mm wide, and 50 mm long as shown in Figure 2b. To test the bending sensitivity of the sensor, a series of curvature support surfaces, ranging in diameter from 40 mm to 90 mm, were 3D-printed. The lower limit of 40 mm was a restriction imposed by the 50 mm length of the sensor. Each support surface included a groove to hold the sensor.

## 3. Results

**Figure 3:**
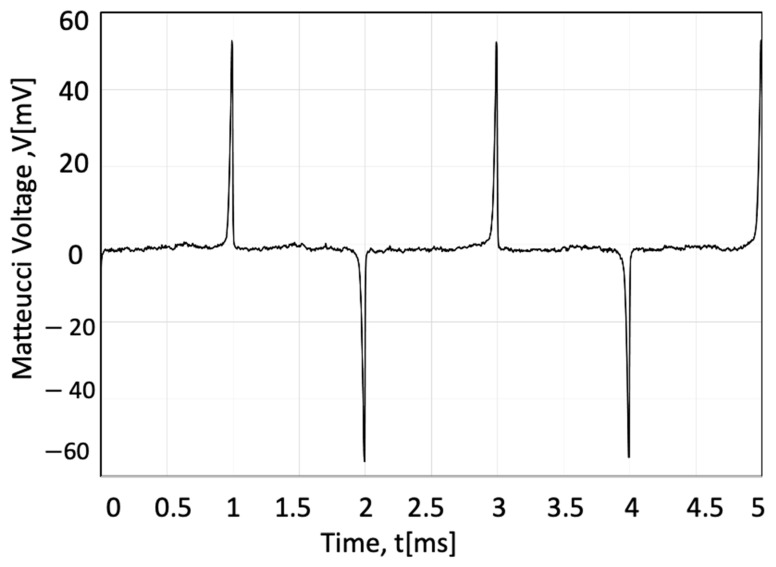
Output Matteucci voltage for the sensor magnetized with 0.9 kA/m magnetic field at 500 Hz frequency twisted 40°/cm (271 MPa torsion stress).

The output Matteucci voltage for an AF10 amorphous wire increases with torsion [39]. Figure 3 shows the Matteucci output measured with an applied 271 MPa torsion stress calculated from the twist angle using the following relationship [41]:

(2) $$\tau =\frac{G\times \theta \times r}{L}\;$$

where *θ* is the twisting angle, *G* is the shear modulus, *r* is the radius of the wire, and *L* is the wire length. For AF10 amorphous wire, *G* was assumed to equal 62 GPa [42]. 

**Figure 4:**
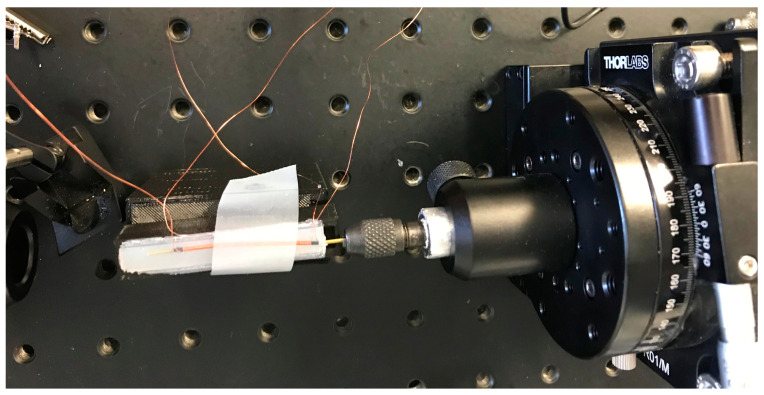
Holding amorphous wire from one end. (Method number one).

Seven sensors were fabricated, four of them (S1–S4) using as-cast amorphous wire and three (SA1, SA2, TSA) using annealed amorphous wire. The fabrication process for S1 and S2 involved constructing a 3D-printed mold with an internal cavity measuring 3 × 5 × 50 mm for embedding the sensor with silicone rubber, i.e., dragon skin 30. During the embedding process, the amorphous wire and solenoid were placed inside the cavity with the wires protruding through holes at the ends of the mold. Sensors S1 and S2 were fabricated by using several stages of silicone embedding. The first stage involved partially embedding the coil and amorphous wire inside a thin silicone rubber layer. In the next stage, a second mold was used in which the wire/solenoid assembly was inserted. One end of the wire/solenoid was pressed against the mold as shown in Figure 4 the other end was left exposed. The whole assembly was coated with a second layer of silicone thereby fixing the exposed wire end. The final stage was to rotate the uncoated wire end by 40°/cm whilst simultaneously embedding the whole arrangement in a third coating of silicone.

**Figure 5:**
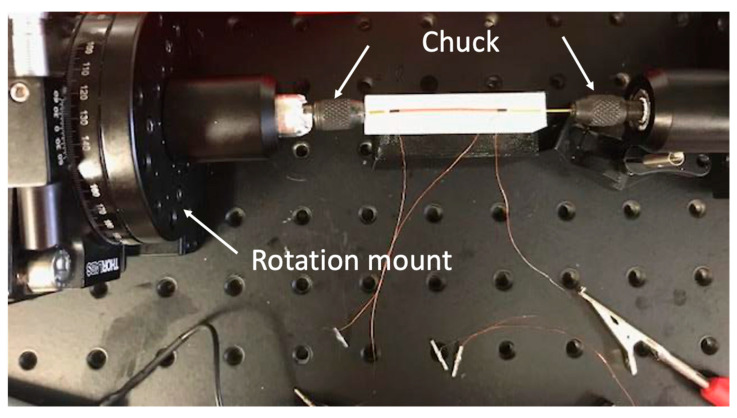
Holding amorphous wire from both sides. (Method number two).

Sensors S3 and S4 were fabricated using an improved method with the ends of the wire connected to chucks to maintain wire straightness whilst applying a twist of 40°/cm as shown in Figure 5.

The mold was filled with silicone rubber and left to solidify for one hour. For the annealed sensors, a 0.5 A current was passed through the wire for one minute. To see the effect of twisting, two of the wires were annealed without any twisting (SA1 and SA2) and the third one was twisted by 40°/cm (271 MPa torsion stress) (TSA). To evaluate the repeatability, measurements were repeated by attaching and removing the sensors from each curved surface ten times. Each set of ten measurements consisted of two groups of five measurements spread over two days to check there were no time-dependent changes such as the untwisting of the wires. The Standard Deviation (SD) of the output Matteucci voltage was calculated using Equation (3).

(3) $$u=\frac{S}{\sqrt{n}}$$

Measurements were taken under ambient room temperature conditions using an oscilloscope to pick up the Matteucci voltage and a micrometer to measure the curvature diameter. The uncertainty budget was calculated based on a SD of 0.25 mV for the oscilloscope resolution and 5 µm for the micrometer resolution. The comparison of the uncertainties, sensitivity, and linearity for all the sensors are summarized in Table 1. The sensitivity is expressed in Equation (4) as the ratio of voltage change divided by the change in radius of curvature (mV/cm).

(4) $$Sensitivity=\frac{\Delta V}{\Delta C}$$

**Figure 6:**
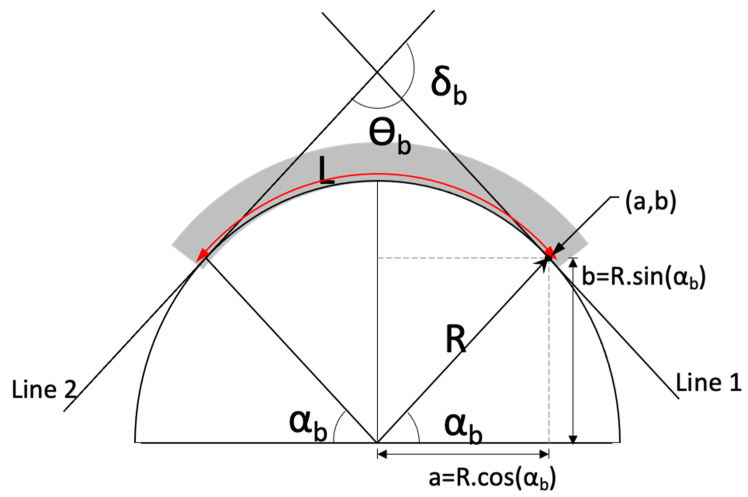
A bending sensor (shaded area) with length *L* following the contour of an arc of radius *R* and bending angle $\delta_{b}$.

As seen in Table 1, the SA1 has the highest sensitivity and linearity, which shows that annealing had a modest effect on improving the sensor’s performance. S4 has the smallest sensitivity because of possible internal stresses in the amorphous wire. The twisted-annealed and annealed sensors (TSA, SA1, SA2) have higher sensitivity in general compared with S1–S4; however, S3 and S2 are comparable with the annealed ones with the highest linearity and sensitivity occurring in SA1. Overall, SA1 is the best sensor, although all sensor performances are comparable except for S4. The bending angle was quantified as the angle between two intersecting tangents (lines 1 and 2) of a curve with radius R as shown in Figure 6. The length of the sensor was fixed and equal to *L*. *R* has a direct relationship with the angle $\delta_{b}$ provided by Equation (5).

(5) $$\delta_{b}=\frac{180\cdot L}{\pi R}$$

Substituting the diameter values (40–90 mm) of the curved surfaces into Equation (5), produces a bending range from 64 to 143°. This was used to characterize the bending sensor in this work. As an example, the uncertainty of the Matteucci voltage measurement was 0.02 mV/cm for SA1. Assuming a linear relation between Matteucci voltage and bend angle, the uncertainty equaled 0.2°.

**Figure 7:**
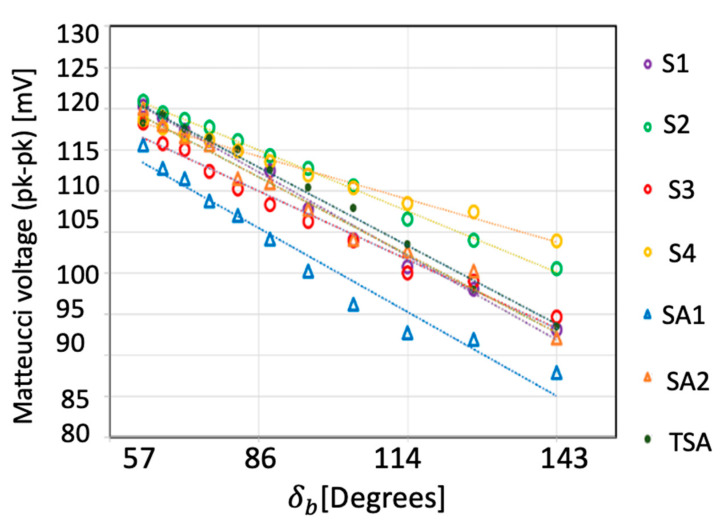
Comparison between sensors with as-cast, annealed, and twisted-annealed AF10 amorphous wire, twisted 40°/cm (271 MPa torsion stress), magnetized in 0.9 kA/m magnetic field and 500 Hz frequency due to bending angle $\delta_{b}$.

As shown in Figure 7, a linear trend line was fitted to each of the sensor outputs against the bending angle. Extrapolating the data in Figure 6 to zero bending angle enables a prediction of the sensor output for a flat surface. A comparison of the predicted values with actual zero bending measurements is shown in Table 2 for all sensors. The maximum difference between the sensor measurement and the extrapolated values is less than 5% suggesting a close linear fit over the whole bending range from 0 to 143°.

## 4. Discussion

**Figure 8:**
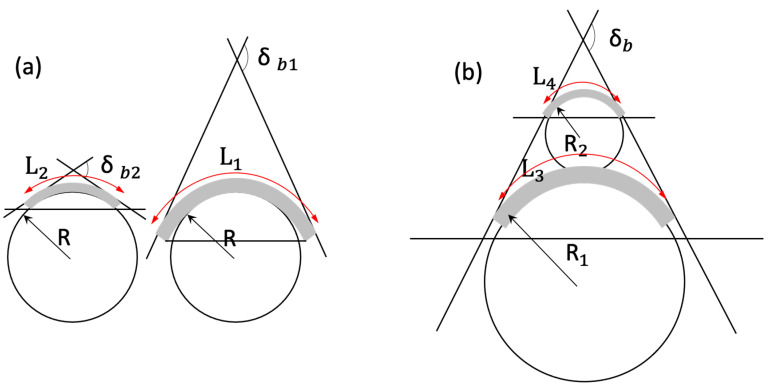
(**a**) The same curvature but different sensor length (shaded area) provides different bend angles; (**b**) different sensor lengths and different curvatures provide the same bend angle.

An important point to note is that the bend angle was defined in terms of the sensor’s length and the radius of curvature of the surface. This is important because special consideration must be provided when calibrating the sensor for specific applications. To illustrate this point, Figure 8 shows two examples of how the sensor’s output voltage depends on both sensor length and surface curvature. Figure 8a shows that for the same curvature, sensors of different length have the same internal stress distribution but different output voltages because the Matteucci effect is also proportional to length. The resultant bending angles are different using the geometrical definition illustrated in Figure 6 despite the curvature being identical. Figure 8b illustrates how two different combinations of sensor length and curvature can provide identical bend angles despite different output values. When measuring bending angle, it is therefore essential that the limitations associated with the sensor’s methodology are fully considered. To summarize, the sensor’s output voltage is inversely proportional to the bending angle (Figure 7) and directly proportional to its length. Therefore, the best signal-to-noise ratio is obtained for large output voltages, when the curvature is small, and the sensor is long. Linearity over small bend angles is an advantage compared with the commercial carbon-based resistive flex sensors [12,43] which are not linear between 0 and 20°and the intrinsically non-linear optical sensors [44,45,46]. Another advantage is its large measurement range of 0 to 143°. This may be extended even further with the use of shorter sensors, although this reduces the output signal level. When using this type of sensor, it is therefore important to calibrate the sensor based on its length and the type of curved surface to be measured. For example, to measure finger flexure the sensor dimensions need to closely match the size of the individual’s joint.

Table 3 summarizes the specifications of some commercially available bend sensors. As seen in the table, most commercial sensors perform with a bend resolution somewhere between 0.1 and 2° and a measurement range from 0 to 90°. However, not all of these exhibit a linear response, the relatively cheap resistive flex sensors for example perform poorly over small bend angles. The sensors developed as part of this work compare favorably with the commercial ones and demonstrated a measurement resolution of 0.2°, a linearity between 0.93 < R^2^ < 0.99 and a measurement range between 0 and 143°.

## 5. Conclusions

To conclude, a flexible and light-weight bend sensor utilizing the Matteucci effect in amorphous wires was developed to measure differences in curvature ranging in diameter from 40 mm to 90 mm. A simple model was proposed in this paper to translate these curvature measurements into equivalent bend angles. The measurement range, in this case, was 64 to 143° but the extrapolation of data to 0 was consistent with a linear output range from 0 to 143°. The sensors are customizable by changing the length of the sensor to suit a particular task. For example, in the case of glove applications, the sensor dimension can be tailored to fit different finger dimensions of children and adults. Seven sensors were fabricated to investigate the variability and repeatability of sensor performance due to manufacturing. Three of them were subjected to annealing during manufacture which improved the linearity of the sensor. The annealed sensors had generally higher sensitivity compared with those untreated, but all sensors exhibited linear behavior. Compared with commercially available flex sensors, the bend sensors developed in this work achieved a better measurement range (0 and 143°) whilst delivering a linearity with an R^2^ value between 0.93 and 0.99, and a sensitivity of 5.68 ± 0.02 mV/cm. A measurement resolution of 0.2° also compared well with the 0.1–2.0° seen in commercial sensors. The feasibility of measuring bend angle using a novel Matteucci effect sensor was clearly demonstrated in this work. Further work is needed to evaluate performance using smaller sensors and to confirm the linearity at small bending angles along with the integration of signal conditioning and power circuitry. The effects of temperature sensitivity and response time on sensor performance also require further investigation.

## Figures and Tables

**Table 1:** Comparison between sensor sensitivity, linearity, and uncertainty when magnetized at 0.6 kA/m, 500 Hz and twisted 0.70 rad/cm (271 MPa torsion stress).

Sensors	Sensitivity (mV/cm)	Linearity (R^2^)	Max SD (mV)	Min SD (mV)	Average SD (mV)	Uncertainty of Sensitivity (mV/cm)
S1	5.45	0.96	0.36	0.18	0.27	0.01
S2	3.95	0.95	0.67	0.24	0.45	0.02
S3	4.45	0.99	0.27	0.11	0.19	0.01
S4	2.79	0.97	0.25	0.07	0.16	0.005
SA1	5.62	0.99	0.61	0.18	0.39	0.02
SA2	5.08	0.96	0.37	0.16	0.26	0.01
TSA	5.12	0.93	0.62	0.33	0.47	0.02

**Table 2:** Comparison between the sensor in flat condition and trendline extrapolation to 0°.

Sensor Type	Flat Condition Output (mV)	Trend Line Extrapolation to 0 (mV)	% Error
S1	137	142.85	4.2
S2	134	137.31	2.5
S3	130	134.98	3.8
S4	130	129.79	0.2
SA1	130	136.11	4.7
SA2	134	140.04	4.5
TSA	136	141.48	4.0

**Table 3:** Comparison of bending sensors.

Sensors	Linearity	Resolution	Measuring Range	Repeatability (SD)	Application
Sensor SA1	0.99	0.2°	0–143°	0.9° < SD < 1.42° (Range for all sensors in this work)	Wearable gloves
Hydrazine-Reduced Porous Graphene sensor [21]	R^2^: 0.99	1°	90°	Mean square error (MSE): 0.105	Blood pressure estimation
Optical sensors [44,45,46]	Non-linear	0.01° < R < 2°	90° to 97°	1.15° < SD < 1.72	Wearable gloves
Flex-resistance sensors [12,43]	Nonlinear between 0 and 20°	R ≤ 2°	90°	0.11° < SD < 1.15°	Wearable gloves

## Data Availability

https://orca.cardiff.ac.uk/id/eprint/131842/ (accessed on 18 December 2022).
